# Development and description of a logic model for a guided, low-intensity cognitive behavioural therapy intervention for parents of persons treated for cancer during childhood: the EJDeR intervention

**DOI:** 10.1136/bmjopen-2026-116503

**Published:** 2026-10-13

**Authors:** Ella Thiblin, Joanne Woodford, Anne H Berman, Paul Farrand, Philip Leissner, Markus Saarijärvi, Louise von Essen, David A Richards

**Affiliations:** 1 CIRCLE – Complex Intervention Research in Health and Care, Department of Women's and Children’s Health, Uppsala University, Uppsala, Sweden; 2 Department of Psychology, Uppsala University, Uppsala, Sweden; 3 Clinical Education Development and Research (CEDAR), University of Exeter, Exeter, UK; 4 Department of Neurobiology, Care Sciences and Society, Karolinska institutet, Stockholm, Sweden; 5 Department of Cardiology, Danderyd Hospital, Stockholm, Sweden; 6 Department of Health and Caring Sciences, Western Norway University of Applied Sciences, Bergen, Norway

**Keywords:** Psychosocial Intervention, Parents, Paediatric oncology, Depression & mood disorders, STATISTICS & RESEARCH METHODS

## Abstract

**Abstract:**

**Background:**

There is a lack of psychological interventions for parents of persons treated for cancer during childhood who report a need for support. We therefore developed a psychological intervention, including a programme theory and logic model as recommended by the UK Medical Research Council. The logic model offers structured ways to articulate how interventions are intended to work and interact within their context. The processes by which programme theories and logic models are developed and refined are rarely described in detail.

**Objective:**

This paper describes the development of the programme theory and logic model of a workbook and audio-administered, telephone-guided, low-intensity cognitive behavioural therapy intervention (EJDeR) for parents of persons treated for cancer during childhood.

**Methods:**

We iteratively developed the programme theory and logic model for EJDeR in line with published guidance, drawing on prior research findings and existing theory. A multistage process involving multidisciplinary researchers and a Parent Advisory Board was used to draft and refine the logic model.

**Results:**

The final version of the logic model outlines intervention resources, intervention delivery, outputs, mechanisms of impact, outcomes and contextual factors.

**Conclusions:**

Using the EJDeR intervention as an example, we have illustrated an iterative, theory and research-informed approach to developing a programme theory and logic model in collaboration with key interest holders, including public contributors. Articulating and refining the programme theory enhanced our understanding of how the intervention is intended to work and provides a transferable example for developing logic models in complex healthcare intervention research.

**Trial registration number:**

The ENGAGE feasibility trial: ISRCTN57233429.

**STRENGTHS AND LIMITATIONS OF THIS STUDY**

- The logic model was developed iteratively and informed by previous research, theory and interest holder feedback.
- Public contribution strengthened the identification of relevant contextual factors.
- Explicit articulation of the logic model clarified key mechanisms through which the intervention is intended to work.
- Programme theory and logic model development were largely conducted within an expert group.
- The lack of systematic documentation of changes made during workshops reduced transparency in the development process.

## Introduction

 Parents of persons treated for cancer during childhood often experience significant psychological challenges. Approximately 350 children are diagnosed with cancer in Sweden each year.^1^ As cancer treatments have advanced, the 5-year survival rate for children and adolescents with cancer in Sweden is now 85%.^2^ During and after treatment, parents are typically the main support for children with cancer, and a diagnosis of childhood cancer is a highly disruptive experience for children and parents. Challenges such as fatigue, depressive symptoms, worry, uncertainty about the future, and feelings of isolation are reported by parents and can persist after completion of the child’s cancer treatment.^3–8^ Across studies, symptoms of depression and anxiety reported by parents ranged from 14.4% to 43.4% and from 19.7% to 43.4%, respectively.^3 7^ Five years after completion of cancer treatment post-traumatic stress symptoms were reported by 7.8% of fathers and 19.1% of mothers.^9^ In Sweden, a need for psychological support is reported by 37% of mothers and 28% of fathers 1 year after treatment completion, and 19% of mothers and 7% of fathers 5 years after treatment.^10^ Difficulties with psychological well-being may affect parents’ everyday life (eg, leisure activities and work).^11^ Furthermore, increased parental psychological distress is associated with greater distress in the child treated for cancer,^12^ and overall family functioning supports healthy psychosocial development in the child.^13^ Therefore, it is important to provide evidence-based care and treatment for parents to improve their mental well-being and, by extension, overall family functioning and the child’s well-being.

In this paper, we describe the development process and present the resulting programme theory and logic model of the EJDeR intervention (Swedish acronym for en arbEtsbok- och ljudbaserad sJälvhjälp för förälDrar till barn som avslutat en behandling mot canceR), developed for parents of persons treated for cancer during childhood who report a need for support. EJDeR is a low-intensity cognitive behavioural therapy (LICBT) intervention with weekly guidance provided by parent guides. LICBT utilises a single-strand approach, meaning that individual evidence-based CBT techniques (eg, behavioural activation for depression) are used in turn, rather than combining multiple techniques.^14^

### Development of the EJDeR intervention and the ENGAGE feasibility trial

Recognising the need for accessible and effective psychological support for parents of persons treated for cancer during childhood, we developed EJDeR. The goal of EJDeR is to improve mental well-being and reduce psychological distress among parents of persons who have completed treatment for cancer during childhood 3 months to 5 years previously, and are in remission. We conducted a series of iterative intervention development studies to inform the development of EJDeR^4 7–11 15–19^ and subsequently tested EJDeR in the ENGAGE study, a single-arm feasibility trial.^6 20–25^ We embedded public contribution throughout the intervention development^17 19^ and feasibility phases.^23 24^ Public contributors in the intervention development phase were four parents (two fathers and two mothers) of persons treated for cancer during childhood,^19^ and in the feasibility phase three former ENGAGE participants (two fathers and one mother)^23 24^ and three parents (two mothers and one father) of persons treated for cancer during childhood who did not participate in ENGAGE. A summary of studies conducted during these phases, leading up to intervention refinements and the programme theory and logic model development, is outlined in figure 1, including preparatory longitudinal work,^9–11^ a systematic review,^4^ a survey on attitudes and preferences towards an intervention^18^ and feasibility testing.^6 20–25^

**Figure 1:**
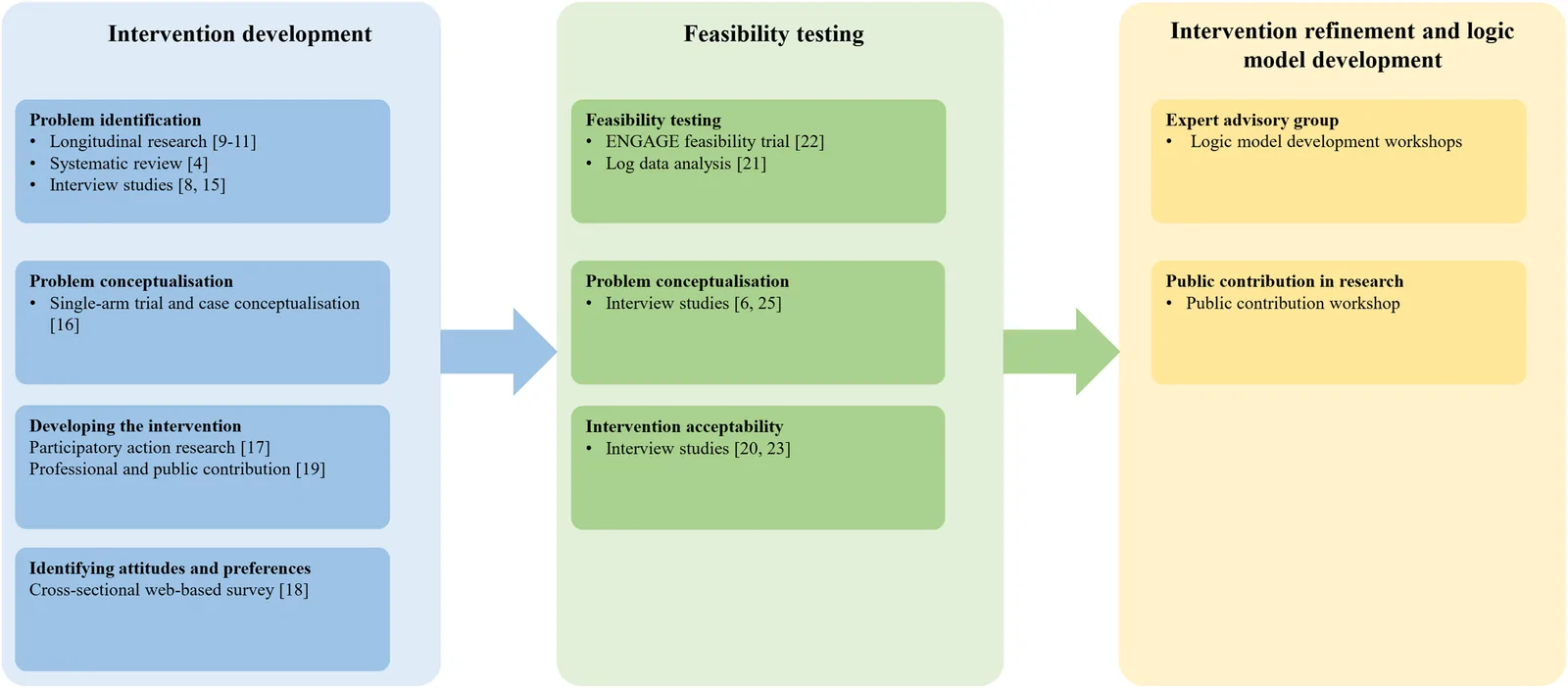
Research informing the development of the EJDeR logic model.

The EJDeR intervention tested in ENGAGE has been described in accordance with the Template for Intervention Description and Replication (TIDieR).^19^ In ENGAGE, EJDeR was delivered online, as an internet-administered CBT intervention. We collected clinical and procedural data and conducted qualitative interviews at baseline to examine parents’ cancer-related concerns and coping strategies.^6 25^ We conducted interviews at post-treatment (12 weeks) to explore the acceptability of EJDeR from the perspective of parents^23^ and parent guides.^20^ Interviews were analysed with qualitative content analysis^26^ and the framework method^27^ (using NVivo software). Results subsequently informed modifications and refinements to EJDeR, for example, including additional psychoeducation and refining intervention language. 72 parents consented and were provided with EJDeR, of whom 47.9% adhered to the minimum treatment dose (defined elsewhere^22^), slightly below the 50% progression criterion.^28^ Parents^23^ and parent guides^20^ reported EJDeR was acceptable and relevant. While results from ENGAGE demonstrated the overall acceptability and feasibility of EJDeR, a need for refinements and modifications was identified and implemented in the iteration of EJDeR described in this paper.

### Programme theory and logic model development

Complex healthcare intervention development, such as the process of developing EJDeR, has been described as a ‘black box’,^29^ where little information on the processes and decision-making involved is reported.^29 30^ This lack of reporting limits others researchers’ possibility to replicate the intervention or conduct subsequent research on it. Furthermore, for researchers to interpret their findings and refine or adapt the intervention accordingly, it is necessary to collect data on how the intervention might work in the contexts in which it might be implemented. One way to deepen our understanding of an intervention beyond simply asking whether it works (ie, evaluating intervention efficacy and effectiveness) is to develop an intervention programme theory and a logic model.^31^ Programme theory development and refinement are core activities recommended in guidance literature across all phases of developing, testing, evaluating and implementing complex interventions.^31–33^

A programme theory provides a roadmap for understanding the mechanisms and contextual factors that influence an intervention’s effectiveness and anticipated outcomes, and articulates the implicit theoretical underpinnings concerning how the intervention might work.^34^ A logic model is a visual representation of the programme theory, presented in a diagrammatic format, typically presenting the following components: intervention resources/input, intervention delivery/activities, intervention outputs,^35^ mechanisms of impact, outcomes and context (see table 1).^36^

**Table 1:** Components of a logic model

Component	Description
Intervention resources/input	Intervention material (eg, workbooks), staff (eg, therapists), technical solutions (eg, online platforms), equipment (eg, telephones) and other resources needed to deliver the intervention.
Intervention delivery/activities	Intervention modality (eg, face-to-face, over the telephone), content offered to recipients and activities undertaken to ensure appropriate delivery (eg, intervention delivery protocols).
Intervention outputs	How recipients enact/engage with the intervention (eg, work with intervention material).
Mechanisms of impact	How intervention components, and recipients’ enactment of them/engagement with them, lead to the intended outcomes (eg, skill development from engaging with intervention material, which leads to better mood and symptom reduction).
Outcomes	What is intended to be achieved from the intervention (eg, symptom reduction, increased mental well-being)
Context	Individual or situational factors (eg, socio-demographic factors, healthcare organisation) that may influence how the intervention works and vice versa (ie, the context may also be influenced by the intervention).

At the outset of intervention development, a programme theory helps identify the proposed active intervention ingredients and how they are implemented. A clear programme theory and logic model can also facilitate communication with funders and other relevant interest holders.^33^ In the feasibility phase, a programme theory helps identify key intervention uncertainties that need to be addressed. In the evaluation phase, identifying mechanisms of change can guide the selection of outcome measures,^37^ including process evaluation measures (eg, measures assessing whether proposed mechanisms of change were related to outcomes^38^). Regarding implementation, an articulated programme theory facilitates adaptation of the intervention to specific contexts.^31^ Throughout all phases, it may help to identify uncertainties and, consequently, inform research questions.^31^ Programme theories and logic models are therefore not static but should be refined throughout the development, test, evaluation and implementation phases.^33^ However, it is rare for the process of developing programme theories and logic models to be described in detail.^39^

### Aims and objectives

This paper aims to describe the development of the programme theory and logic model for a workbook and audio-administered, telephone guided, LICBT intervention (EJDeR) for parents of persons treated for cancer during childhood.

## Methods

We developed the programme theory and logic model for EJDeR iteratively in accordance with the UK Medical Research Council (MRC) framework for the development and evaluation of complex healthcare interventions.^31^ We used findings from the intervention development and feasibility phases (see figure 1) to develop the programme theory and logic model, outlining: (1) intervention resources/input; (2) intervention delivery/activities; (3) intervention outputs; (4) mechanisms of impact; (5) outcomes; and (6) context.

### Procedure for programme theory and logic model development

We developed the programme theory, drafted the associated logic model and refined it in four stages informed by interest holder feedback.

#### Stage one

We held six online intervention development workshops (≈60 min) attended by a multidisciplinary team: authors ET (licensed psychologist, PhD candidate), JW (PhD, LICBT expert, expert in complex interventions in healthcare research), PF (PhD, LICBT expert, expert in complex interventions in health services care research) and AHB (licensed psychologist, PhD, iCBT expert). During these workshops, we presented and discussed drafts of the logic model, informed by findings from the feasibility phase.

#### Stage two

We held four additional online workshops (≈60 min) attended by authors DAR (registered nurse, psychological therapist, PhD, expert in complex interventions in health services research), MS (registered nurse, PhD, expert in complex interventions in health services research), JW and LvE (licensed psychologist, PhD, expert in complex interventions in healthcare research, project principal investigator who oversaw programme theory and logic model development) to refine the programme theory and logic model developed during stage one.

#### Stage three

We held an online workshop (2 hours) with a Parent Advisory Board (PAB), attended by four fathers and four mothers of persons treated for cancer during childhood (public contributors) and coordinated by one research team member and one father of a person treated for cancer during childhood (coordinators). PAB members were presented with the logic model and asked to provide insight primarily on potentially important contextual factors.

#### Stage four

Final revisions to the logic model were done by DAR, ET, JW and LvE, and we finalised the logic model in June 2025. In the final logic model, key intervention resources and inputs, as well as intervention delivery/activities, were specified and the rationale and theory underlying them were articulated. We formulated a clear description of the expected intervention outputs, the theoretically informed mechanisms of impact and their interaction. Next, we identified outcomes expected from the intervention and measures used to assess them, including process evaluation tools.

#### Patient and public involvement

Public contributors were involved in the development of the programme theory and logic model, see paragraph Stage three.

## Results

### Logic model development

An initial logic model, developed during stage one, is shown in figure 2. This stage was conducted in parallel with refining the therapeutic content of EJDeR, focusing on intervention components and their potential evaluation, rather than intervention delivery. Accordingly, we used a logic model typology to outline intervention components, mechanisms of change and outcomes.

**Figure 2:**
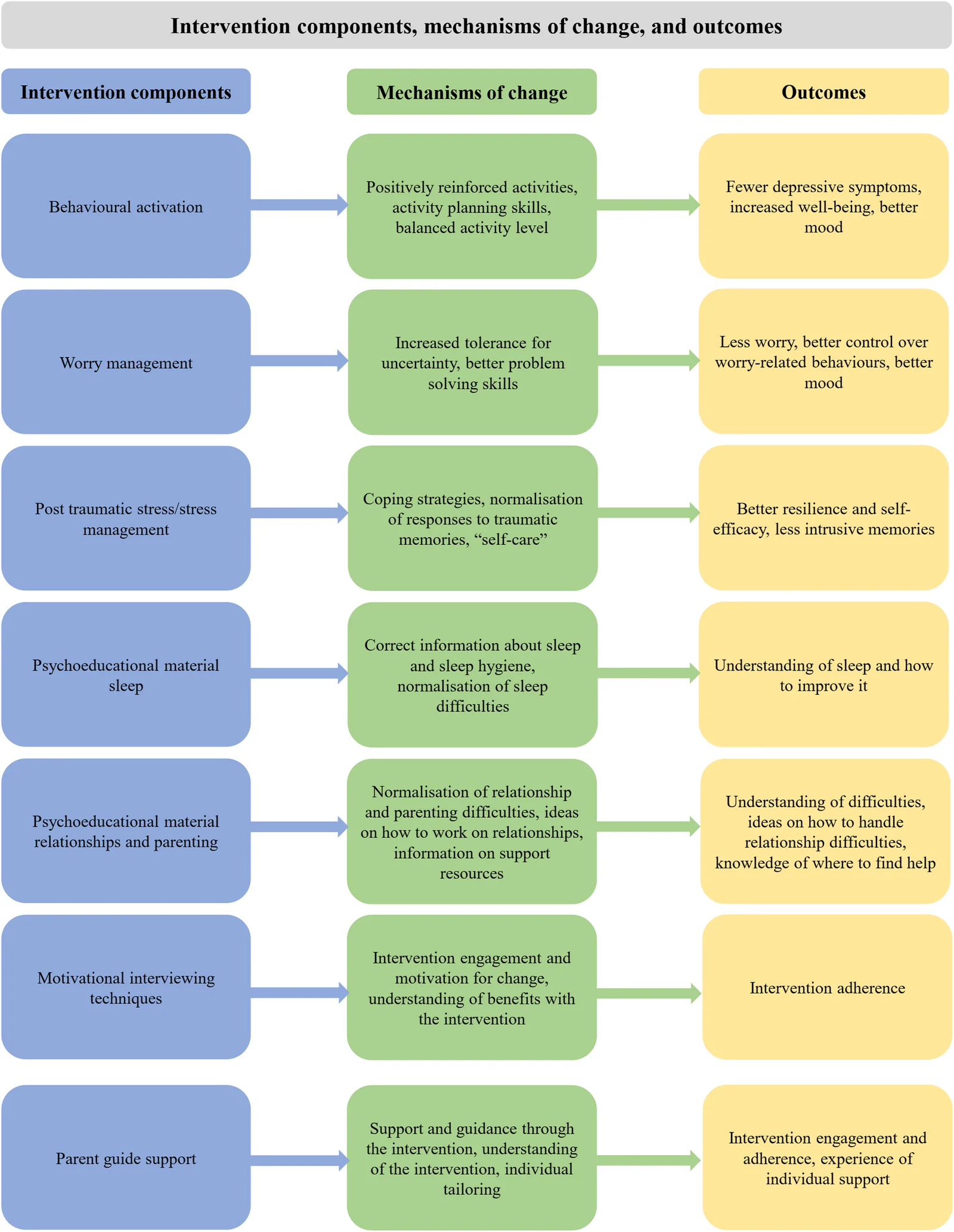
Initial version of the EJDeR logic model, focusing on intervention components, mechanisms of change and outcomes.

An intermediate version of the logic model is shown in figure 3. During this stage, we decided to use a logic model typology outlining intervention resources/input, intervention delivery/activities, intervention outputs, mechanisms of impact, outcomes and context. This iteration was found to be too complex and detailed for effective communication with interest holders outside the research team.

**Figure 3:**
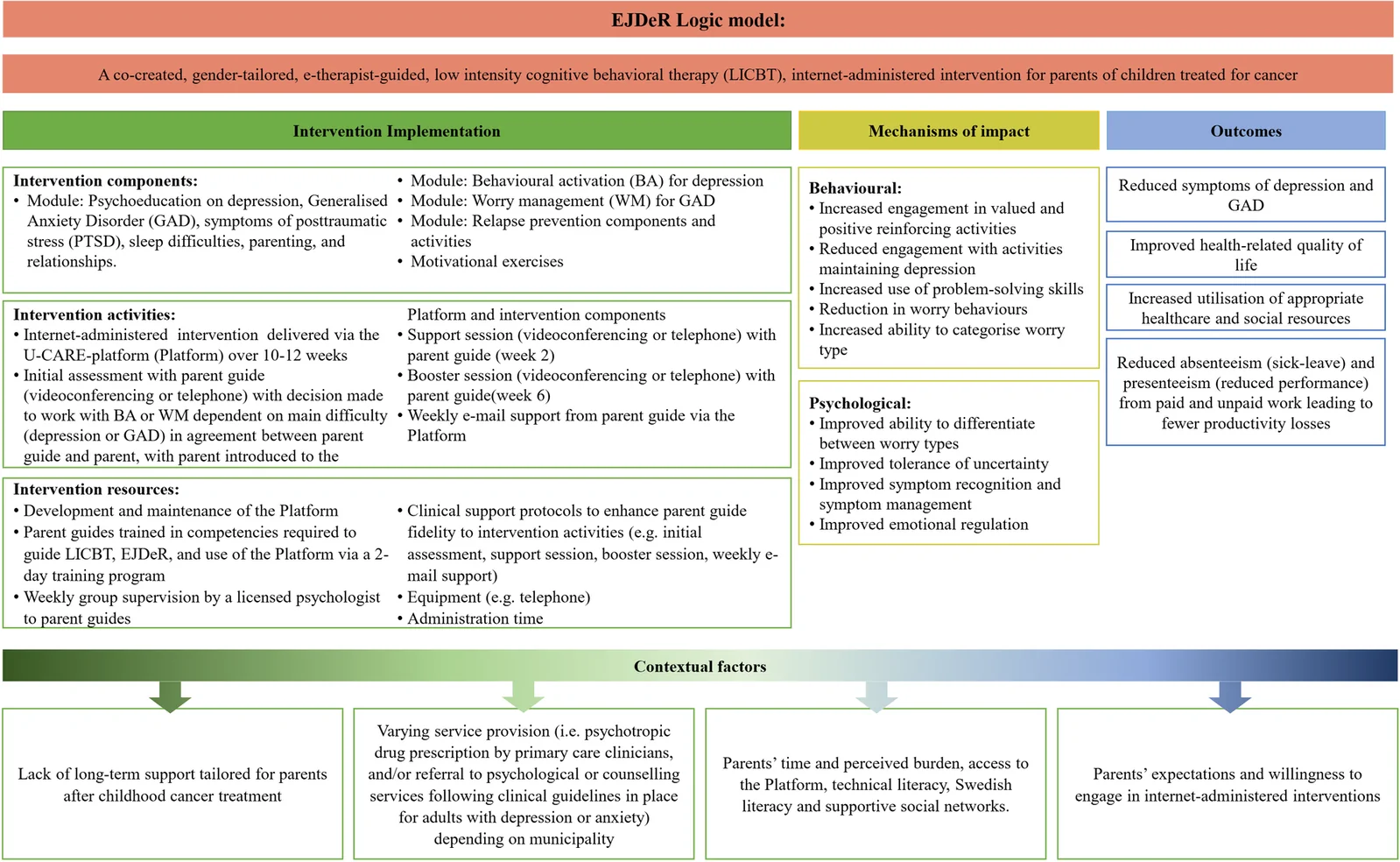
Intermediate version of the EJDeR logic model, with the logic model outlining intervention resources/input, intervention delivery/activities, intervention outputs, mechanisms of impact, outcomes and context. EJDeR logic model as planned for now. Revisions may be made after further collaboration with Parent Advisory Board and experts in LICBT.

The final logic model is shown in figure 4. It has a simpler structure, and includes input from public contributors, for example, the mode of delivery was adapted to provide a workbook and an audio-administered version of EJDeR, and contextual factors, for example, the child’s diagnosis and treatment, and parents’ prior experiences with healthcare were added.

**Figure 4:**
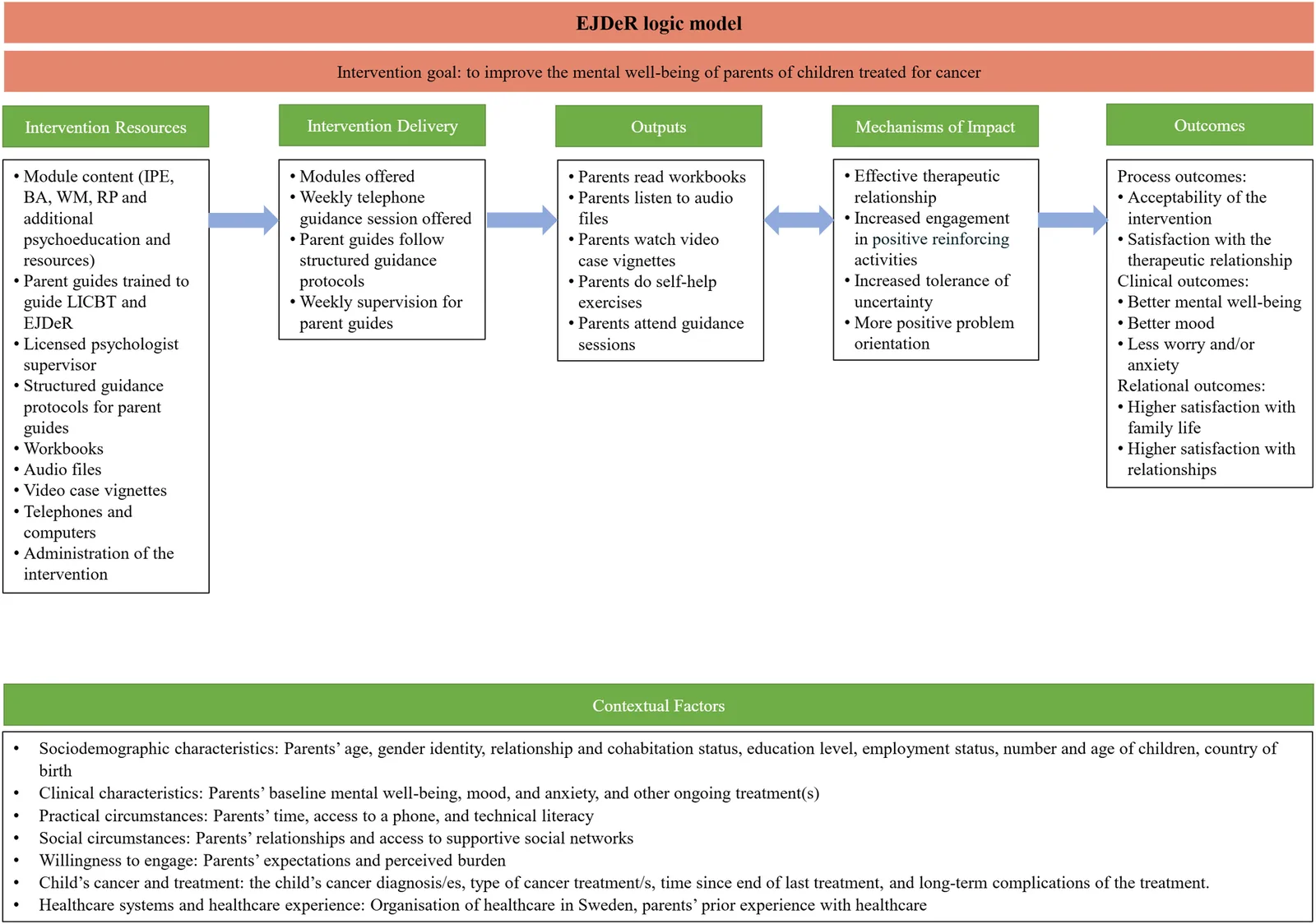
Final version of the EJDeR logic model. BA, behavioural activation; IPE, introduction and psychoeducation; LICBT, low-intensity cognitive behavioural therapy; RP, relapse prevention; WM, worry management.

### Description of the EJDeR programme theory and logic model

We further describe the results (ie, the EJDeR programme theory and logic model) according to intervention resources/input, intervention delivery/activities, intervention outputs, mechanisms of impact, outcomes and context.

#### Intervention resources

##### Module content

Intervention resources include five modules: (1) introduction and psychoeducation, (2) behavioural activation, (3) worry management, (4) relapse prevention and (5) additional psychoeducation and resources. Modules include psychoeducational text, LICBT exercises and case vignettes with parents of persons treated for cancer during childhood in text and video format.

Introduction and psychoeducation: Parents are introduced to EJDeR and the five areas CBT model (ie, situation, thoughts, emotions, physical symptoms and behaviours).^40^ Parents map their current problems to the five areas CBT model, set goals and choose the most appropriate LICBT technique (behavioural activation or worry management) based on their main presenting problem. Information on strategies for maintaining motivation is provided, and parents are encouraged to identify strategies for managing periods of low motivation. Case vignettes, presented in video and text formats, of two parents presenting with low mood and worry, respectively, are included to promote recognition among parents and to support their selection of the most appropriate LICBT technique.

Behavioural activation: The module is informed by brief behavioural activation, as described in established LICBT protocols.^14^ Behavioural activation is a well-established, evidence-based treatment approach for depressive symptoms.^41 42^ It may prevent symptom development, reduce subclinical symptoms of depression and promote well-being across populations,^43–45^ and it is effective when delivered in an LICBT format.^46^ Behavioural activation works by encouraging people with depressive symptoms to re-engage with valued positive environmental stimuli and reduce the negatively reinforced avoidance of potentially aversive situations. Behavioural activation is individualised through the identification of depressed behaviours, analysis of triggers and behavioural consequences and the scheduling of positively reinforcing goal-oriented activities.^41 42^ In our previous research,^8 16^ parents reported low mood and lack of energy, which fits well with established cognitive behavioural conceptualisations of depression.^47^ Our previous research shows that during the child’s cancer treatment, contact with positive reinforcers naturally decreases while parents care for the child and engage with cancer treatment services.^6 16^ During the treatment period, focusing on the child’s health is, of course, necessary. However, if this behavioural pattern continues after treatment completion, a vicious cycle of avoidance (the target of behavioural activation) can be established, leading to low mood. The behavioural activation module uses words such as ‘feeling down’ and ‘lacking energy’ rather than ‘depression’ and ‘depressive symptoms’ as it is hypothesised to be helpful for both parents reporting depression and parents with subclinical symptomatology.

Worry management: The module comprises two LICBT techniques: worry time for hypothetical (future-oriented) worry and problem solving for practical problems, as described in established LICBT protocols.^48^ Fear of recurrence and persistent worry about the child’s future health are reported by parents^5 6 49^ and can be conceptualised as future-oriented worry, driven by intolerance of uncertainty (ie, finding it difficult to accept the possibility of a negative event occurring^50^). Parenting behaviours reported by parents, such as vigilance for symptoms of illness in the child, overprotecting the child and insisting on a strictly healthy lifestyle,^5 6 8^ can be conceptualised as cognitive avoidance of distressing emotions. They may also serve as a way for parents to regain a sense of control in an uncertain situation.^51^ Informed by ENGAGE findings, the worry management module is tailored to the population by addressing cancer-specific worries. While parents’ worries can be understood through a generalised anxiety disorder (GAD) lens, cancer-related worries may have a different focus than those typically seen in GAD, where worries tend to cover a variety of different life areas. Since cancer-related worries stem from a genuinely catastrophic experience, these worries may not feel excessive to the parents, even if persistent. To tailor the module to parents, exercises include categorising worries as cancer-related or not cancer-related. Additionally, the module provides psychoeducation about the difference between worry about current and (hypothetical) future events, and dwelling on past experiences. Like the behavioural activation module, the worry management module does not explicitly mention GAD, but instead uses wording such as ‘being worried’ and ‘feeling tense’ as it is hypothesised to be helpful for both parents with anxiety and parents with subclinical symptomatology.

Relapse prevention: The module supports the parent in summarising their work with EJDeR and planning to continue it using helpful techniques. Parents identify potential warning signs using the five areas CBT model^40^ and are encouraged to ‘check in’ with themselves regularly on how they are doing and, if necessary, apply LICBT techniques. Informed by ENGAGE findings that video content was appreciated^23^ and by public contributors’ suggestions to adopt a more positive tone, this module includes two video case vignettes featuring parents’ recovery stories.

Additional psychoeducation and resources: The module was informed by ENGAGE findings that parents struggle with challenges not fully addressed by behavioural activation and worry management^6 23^ and includes psychoeducation on motivation, relationships to other adults, parenting, common reactions to traumatic events, alongside imagination self-help exercises to help with emotion regulation, sleep, as well as signposting to relevant resources for parents of persons treated for cancer during childhood. Psychoeducation on motivation, together with brief motivational exercises grounded in motivational interviewing,^52^ is included to improve adherence to EJDeR and is intended for use as needed.^53^

As ENGAGE participants expressed difficulties engaging in social relationships, not limited to the avoidance of activities common in depression,^6^ psychoeducation on relationships with other adults is included. Normalising these relationship difficulties and providing brief advice on navigating them in psychoeducational material may enhance the perceived relevance of EJDeR and motivate parents to re-engage with social relationships and use social support. Increased social engagement may facilitate engagement with LICBT content, for example, by incorporating social activities into behavioural activation.

ENGAGE participants^23^ and public contributors reported that EJDeR lacked a family and parenting perspective. Based on this, psychoeducation on parenting is included, addressing common challenges faced by parents of persons treated for cancer during childhood.^6^ For example, ENGAGE participants expressed an urge to overprotect their child, and uncertainty about distinguishing typical child development and behaviours influenced by the cancer experience, making it difficult to know what demands are appropriate to put on the child.^6^ Acknowledging parenting challenges is included to strengthen the perceived relevance of EJDeR and help parents reflect on parenting behaviours.

The prevalence of post-traumatic stress disorder (PTSD) in parents of persons treated for cancer during childhood does not differ from the general population^54^; however, parents describe avoiding thinking about the cancer experience, and places and situations that remind them of their child’s illness.^6 8^ Emotional and experiential avoidance are core maintaining factors in PTSD, and parents who engage in avoidant coping strategies may be at risk of developing symptoms of PTSD.^55^ Psychoeducation on common reactions to traumatic events and visualisation self-help exercises to help with emotion regulation are included for parents who struggle with intense emotional reactions when reminded of the cancer experience,^56^ and may help increase the acceptability of EJDeR. However, parents with severe symptoms of PTSD are encouraged to speak to their parent guide, who can signpost to more extensive support than EJDeR can provide.

Psychoeducation on sleep is included, as some parents report sleep disturbances resulting from worry and rumination about their child’s cancer and treatment.^6 8^ A list of national resources is provided for parents who require additional support for themselves, their child, or their family beyond what EJDeR can offer. This includes financial counselling, family counselling and child psychiatric services.

##### Workbooks, audio files and video case vignettes

EJDeR is mainly delivered via printed workbooks and audio files. The introduction and psychoeducation and relapse prevention modules also include video case vignettes depicting parents working with EJDeR. The workbook format is well established within LICBT,^14^ and careful attention was paid to workbook design to enhance parents’ willingness to engage with the material. EJDeR workbooks were designed in collaboration with a professional graphic design company to make the workbooks visually appealing and easy to navigate. Illustrations and case vignettes depict parents from diverse ethnic backgrounds and in different family constellations.

Audio files accompany all workbook material, recorded by voice actors and the main actor, who reads most of the material, is also a licensed psychologist. The decision to provide EJDeR in an audio format was informed by public contributors who suggested providing EJDeR as audio files rather than online may enhance intervention flexibility and engagement. In addition, feedback from participants^23^ and parent guides^20^ in ENGAGE indicated that parents experienced technical difficulties and perceived the online format as not user-friendly. Parents also reported being tired of spending time at computer screens, especially after the increased work-related screen time associated with the COVID-19 pandemic.^23^ The use of digital and technical solutions in the workplace can contribute to stress and anxiety,^57^ and excessive screen time has been linked to depression.^58^ Offering alternatives to online self-help may increase acceptability and engagement, and it may be beneficial to provide multiple delivery options for parents to choose based on their preferred learning style.^59^

##### Other intervention resources and input

Other resources include parent guides, licensed psychologist supervisor and parent guide support protocols, whose functions are described under intervention delivery. Parent guides are healthcare workers (eg, psychologist, nurse) with CBT competence who are provided with training in LICBT, EJDeR, and the specific needs and concerns experienced by parents of persons treated for cancer during childhood (including information on different cancer diagnoses and treatments). Furthermore, technical equipment (eg, computers, telephones) and the administration of the intervention (eg, resources for scheduling guidance sessions and posting workbooks to parents) are additional intervention resources.

### Intervention delivery

EJDeR includes (1) parent guides offering modules to parents; (2) weekly telephone guidance by parent guide offered to parents; (3) parent guides following structured support protocols; and (4) weekly supervision for parent guides.

#### Parent guides offering modules to parents

Parents are instructed to begin using the introduction and psychoeducation module on receiving the EJDeR workbooks and audio files. During subsequent weeks, parents are offered the remaining modules after discussing their main presenting difficulty with their parent guide. Parents may choose to work with the behavioural activation or worry management module, or both. However, because EJDeR is a single-strand intervention, parents are encouraged to work with only one module at a time and may use the same LICBT technique for several weeks. All parents are offered the relapse prevention and additional psychoeducation and resources module.

#### Weekly telephone guidance with parent guide

Telephone guidance is included as guided LICBT for depression and anxiety shows larger effect sizes than unguided.^60 61^ Furthermore, some evidence suggests human support may be more important for the success of self-help CBT interventions than any specific technique.^62^ Guidance is provided over the telephone, consistent with ENGAGE findings,^23^ which showed parents preferred telephone contact to written parent guide communication. During the first week of the intervention, parents are offered an assessment session (approximately 45 min) during which their main presenting problem is identified in collaboration with the parent guide. In subsequent weeks, check-in sessions (approximately 15 min) are offered, during which parent guides provide feedback on the parent’s work with the intervention, problem-solve challenges and plan for the upcoming week. During the last week, parents are offered a relapse-prevention session (approximately 30–45 min) in which their work with EJDeR is summarised and a relapse-prevention plan is developed. To promote attendance, guidance sessions are encouraged to be held on the same day and time each week.^63^ Guidance sessions can be understood through the model of supportive accountability,^64^ which suggests the presence of a benevolent, competent and trustworthy ‘coach’ enhances intervention adherence. A positive experience of accountability requires clear roles and articulated expectations regarding the treatment and the relationship, with both parties actively engaged.^64^ Building trust in telephone-administered LICBT relies on the therapist’s ability to listen actively and convey empathy without non-verbal cues.^65^ To ensure this, parent guide training includes materials and exercises focused on developing therapeutic skills for telephone-administered LICBT.

#### Parent guides follow structured guidance protocols

Guidance protocols in EJDeR are informed by established LICBT protocols,^66^ adapted for different sessions (eg, assessment, check-ins and relapse prevention). In line with the model of supportive accountability,^64^ empathy, role clarification and collaborative work are emphasised in EJDeR guidance protocols and parent guide training. Including structured guidance protocols may also enhance parent guides’ fidelity to the intervention protocol, which is associated with better outcomes.^67^ Balancing fidelity to structured protocols with personalised contact can be challenging, and telephone guidance may be perceived as impersonal.^68^ To mitigate this risk, support protocols instruct parent guides to ask open-ended questions and to allow parents to lead discussions in their own words. EJDeR guidance protocols also clarify the necessity of between-session self-help exercises and provide a structure for follow-up.

#### Weekly supervision for parent guides

Parent guides receive supervision from a licensed psychologist to support clinical decision-making and enhance LICBT skills. Supervision supports parent guides’ fidelity to the LICBT protocol and prevents drift toward high-intensity CBT approaches.^69^

### Outputs

In EJDeR, intervention outputs include parents’ enactment of EJDeR: reading workbooks, listening to audio files, watching video case vignettes, attending guidance sessions with their parent guide and doing self-help exercises between sessions.

#### Reading workbooks and listening to audio files

Parents can choose to engage with EJDeR content through workbooks, audio files or a combination of both. They are expected to carry out self-help exercises throughout the modules.

#### Attending guidance sessions with parent guide

During guidance sessions, parents receive help with problem-solving difficulties they encounter and have time to discuss the LICBT rationale and techniques in depth with their parent guide. Parent guides also discuss the rationale for between-session engagement in self-help exercises with the parent, a core characteristic of CBT^70^ and particularly important in LICBT, where therapist time is limited.^71^

#### Doing self-help exercises between sessions

Between-session engagement with self-help exercises is associated with better treatment outcomes^72^ and is characteristic of successful CBT self-help interventions.^62^ Despite this, engaging patients in between-session self-help exercises is a common challenge in telephone-administered CBT,^73^ including LICBT.^71^ Positive beliefs about the helpfulness of between-session exercises enhance engagement and can be facilitated by parent guides who are trained in planning and providing rationale for exercises, following-up on specific exercises and praising effort and engagement.^70^

### Mechanisms of impact

EJDeR is proposed to lead to desired outcomes through (1) general therapeutic mechanisms (ie, an effective therapeutic relationship), and (2) CBT-specific mechanisms (ie, increased engagement in valuable activities, increased tolerance of uncertainty and a more positive problem-orientation).

#### General therapeutic mechanisms

The role of common factors in psychotherapy (eg, factors common across most therapy types), including the quality of the therapeutic relationship, remains a topic of debate and their contribution to treatment outcomes is not fully understood.^74^ In CBT, the general stance is that the therapeutic relationship is necessary, but not sufficient for the therapeutic effect.^75^ From a recipient perspective, contact with a therapist is commonly reported as an important factor in intervention acceptability and success, as reported in LICBT settings.^23 76^ Agreement on treatment goals, agreement on tasks and bond between therapist and client are all factors associated with favourable treatment outcomes,^77^ and guidance in EJDeR from a parent guide is designed to promote a high-quality therapeutic relationship.

#### CBT-specific mechanisms

Behavioural activation is hypothesised to improve mood by disrupting patterns of negatively reinforced avoidance and increasing exposure to positively reinforcing stimuli in a structured, graded manner.^47 78^ Worry time, practised in the worry management module, instructs the parent to schedule worry for a specific time every day and refocus when worrying thoughts occur at other times.^51^ Limiting time spent on worrying during the day gradually increases exposure to living with uncertainty without attempting to control it. Problem-solving, which is also part of worry management, provides a structured approach to identifying and evaluating solutions, even when the outcome of the attempt is uncertain. Practising problem-solving increases confidence in problem-solving ability and fosters a more positive problem orientation.^51^

### Outcomes

The intended outcomes of EJDeR relate to intervention processes (ie, process outcomes), mental well-being and symptoms (ie, clinical outcomes) and important relationships (ie, relational outcomes). We have identified potential outcome measures to assess intended intervention outcomes.

#### Process outcomes

Intervention acceptability can be understood as a process-related outcome, as engagement with EJDeR should ideally lead to an experience of EJDeR being relevant, coherent and helpful. The retrospective version of the Theoretical Framework of Acceptability (TFA) questionnaire can measure intervention acceptability after EJDeR completion,^79^ and qualitative interviews informed by the TFA can further explore mechanisms of impact. An effective therapeutic relationship is a proposed mechanism of change in EJDeR. The Working Alliance Inventory^80^ can be used to assess this outcome, and qualitative interviews can further explore it.

#### Clinical outcomes

Improved mental well-being is the proposed key outcome for EJDeR, and, given the target population does not necessarily present with clinical diagnoses, a measure of mental well-being may be more sensitive to change than diagnosis-specific measures. Examples of outcome measures include the Questionnaire on Well-Being^81^ and the Warwick-Edinburgh Mental Well-being Scale.^82^

EJDeR targets symptoms of depression and worry, and symptom scales are therefore relevant to use in intervention evaluations. The Patient Health Questionnaire-9 (PHQ-9),^83^ Patient Health Questionnaire-8 (PHQ-8)^84^ and GAD-7^85^ are commonly used to evaluate LICBT interventions and are suitable for measuring outcomes at multiple time points because they are brief and easy to administer. Given parents of persons treated for cancer during childhood often present with symptoms resembling PTSD, and that EJDeR includes strategies to deal with reactions to traumatic events, it is relevant to measure PTSD symptoms, for example, with the PTSD Checklist - Civilian Version.^86^

#### Relational outcomes

Increased satisfaction with family life can also be expected, given the interrelatedness of parents’ and children’s well-being.^12^ As parents’ well-being improves, their relationships with their child and other family members may also improve. This outcome can be explored, for example, through qualitative interviews.

### Contextual factors

Drawing on our previous research, feasibility testing, collaboration with public contributors and the wider literature, we identified both individual-level and systemic contextual factors that may influence how EJDeR works. These include socio-demographic characteristics, clinical characteristics, practical circumstances, social circumstances, willingness to engage, the child’s cancer and treatment and healthcare systems and healthcare experience.

#### Socio-demographic characteristics

Sex or gender may affect willingness to work with EJDeR, as women are more likely to seek professional care for mental health issues.^87^ Men being less likely to seek help for mental health problems compared with women has been attributed to stigma and masculinity norms.^88^ Our previous research shows that mothers of persons treated for cancer during childhood are at higher risk for developing PTSD than fathers,^9^ and other research groups have found significantly higher levels of dysfunctional fear of cancer recurrence in mothers,^49^ potentially pointing to gender differences in support needs. However, our research has also shown that childhood cancer has a greater impact on fathers’ long-term financial situation than mothers’^89^ and more fathers were on sick leave at 5 years than at 1 year after the end of cancer treatment/or a child’s death.^90^ These findings indicate a complex relationship between parental gender and long-term adjustment after childhood cancer.

In the Swedish context, migrants have a different pattern of help-seeking behaviour regarding psychiatric care than Sweden-born citizens, as migrants tend to underuse psychiatric care during their first decade in Sweden, but increase their utilisation with time.^91^ Reasons for underutilisation are not known, but research from other countries, such as the UK, points to cultural factors, for example, illness perception and stigma, as an influence on willingness to seek help for mental health problems among ethnic minorities.^92^ Socio-demographic factors can also affect how the intervention is perceived and how effective it is. For instance, higher education levels are associated with better treatment outcomes in guided self-help CBT.^93^

#### Clinical characteristics

Parents’ mental well-being and symptoms of psychological distress at the start of the intervention may influence the intervention’s relevance and effectiveness, for example, absolute response rates in therapy increase significantly with higher baseline depression severity.^94^ Some proposed mechanisms of change relate to low mood and worry and may not apply to parents who do not experience these symptoms.

#### Practical circumstances

Although the intervention is low in technical complexity, parents must have access to a computer, tablet or smartphone to access audio and video content.

#### Social circumstances

Having access to supportive networks, for example, living near friends and relatives who can assist with everyday responsibilities, may facilitate intervention engagement.

#### Willingness to engage

Prospective acceptability, for example, perceptions of intervention burden, relevancy and effectiveness,^95^ may affect willingness to work with the intervention. Some parents may view guided self-help as ‘second best’, as some recipients report preferring face-to-face interventions over telephone-administered.^65^ ENGAGE participants voiced both positive and negative views of internet-administered treatments, and some limitations mentioned, for example, requiring self-discipline and lack of personal contact^23^ may be considered applicable to self-help interventions in general.

#### Child’s cancer and treatment

The child’s cancer diagnosis, treatment type and time since treatment may be relevant contextual factors. Some research indicates cancer type may affect parents’ functioning after the end of treatment, for example, diagnoses and treatments with more severe late effects are associated with lower parental functioning and may be linked to greater fear of recurrence in parents.^49 96^ However, findings on the association between illness-related variables and parental adjustment are mixed: survey studies have demonstrated no link between cancer-related variables and parental quality of life,^3 97^ and the effect of time since the child’s diagnosis and treatment on parents’ adjustment is inconsistent.^4^

#### Healthcare systems and healthcare experiences

In Sweden, six paediatric oncology units are distributed over the country. During treatment, psychosocial care is offered to the whole family, and parents are generally satisfied with the support they receive.^6^ After treatment completion, parents and children attend follow-up visits during which the family’s well-being is addressed. The paediatric oncology unit may offer parents support from counsellors for a limited period.^98^ However, parents in need of more extensive support are referred to the general healthcare system, which in Sweden is organised into 21 decentralised regions, following the same general guidelines but independently structuring and organising healthcare.^99^ Currently, no tailored support for parents of persons treated for cancer during childhood is offered, and competency around the population’s needs may vary across the country. The Swedish Childhood Cancer Fund (Barncancerfonden) provides support groups, organises activities for families and plays an important role in supporting families affected by childhood cancer. It is, however, not their role to provide evidence-based psychological support for parents. Parents often experience the transition between the child’s treatment period and life after as difficult, as it is not clear where to turn to with their own psychological needs.^6^

## Discussion

We have described the development of the programme theory and logic model for a workbook and audio-administered, telephone guided, LICBT intervention (EJDeR) for parents of persons treated for cancer during childhood. The logic model includes intervention resources/inputs, delivery/activities, outputs, mechanisms of change (ie, the pathways through which EJDeR is intended to work), the intended outcomes of EJDeR, suggested measures to assess these outcomes, and relevant contextual factors for the intervention. The development of the EJDeR logic model was iterative, and it may be further refined based on future research. This iterative development approach has been used in previous research^39 100^ and is preferable because it enables a reflective process during development, thereby approximating a more realistic logic model. The EJDeR logic model is an effective example of how the development of such a model can be informed by both theory and research, in conjunction with interest holder feedback, including public contributors.

### Theoretical assumptions

With the EJDeR logic model, we aim to make explicit the theoretical assumptions that are often taken for granted in intervention development and evaluation research. However, it was challenging to discuss and critically evaluate all relevant assumptions in the logic model format. In our case, the fundamental conceptualisation of parents’ problems could be called into question. Conceptualising parents’ cancer-related psychological distress as something that can and should be met with a healthcare intervention is associated with certain assumptions and ideas that may be specific to the cultural context and research paradigm. CBT is grounded in Western values^101^ and emphasises change at the individual level. From a CBT perspective, an individual’s thoughts, feelings and behaviours are often labelled as dysfunctional, implying the solution lies in the person’s active effort to change how they act and think, and, consequently, how they feel. However, when considering the experience of being a parent to a person treated for cancer during childhood, this framework may not fully apply. For some parents, an existential, religious or spiritual perspective^102 103^ may more accurately reflect their lived reality. In such cases, support from informal or faith-based communities may be more meaningful than seeking help within the healthcare system. Our example may illustrate a limitation of developing logic models within ‘expert groups’ in which interest holders share similar beliefs and experiences regarding a particular treatment, such as CBT.

### Implications for future evaluations

Contextual factors described in the logic model may have important implications for future evaluation and application. For example, the role of socio-demographic factors for the acceptability and effectiveness of EJDeR deserves attention. Women were overrepresented in ENGAGE,^22^ mirroring the overall distribution of gender among individuals seeking professional care for mental health problems.^87^ Additionally, the education level of ENGAGE participants was higher than in the general population in Sweden,^22^ which may have led to an overestimation of the overall acceptability of EJDeR in parents of persons treated for cancer during childhood, as education level is positively correlated with beneficial outcomes in guided, self-help CBT.^93^ Despite efforts to recruit broadly, ENGAGE also failed to attract participants from diverse ethnic and cultural backgrounds.^22^ Future evaluation should pay close attention to recruitment practices; for instance, the SupporTing Recruitment and retention Improvements for Diverse Ethnicities (STRIDE) recommendations^104^ can be used to strengthen inclusivity in trials. Considering such contextual factors is a crucial step before moving on to implementation. Specifically, regarding the EJDeR intervention, future studies could address these questions by employing a mixed-methods process evaluation design.^38^ When assessing the feasibility of adapting logic models to new contexts, existing guidelines, such as the Adapting interventions to new contexts (ADAPT) guidance,^105^ can support decision-making.

### Implications for implementation

Most interventions are developed with the ambition of a future implementation. However, implementation is difficult and should be considered from the outset of intervention design.^106^ The most recent MRC framework^31^ advises considering future implementation through all phases of intervention development and evaluation. One commonly recommended component for facilitating implementation is the development of a logic model.^106 107^ Although a logic model is a helpful tool for facilitating implementation, there are logic model templates specifically designed for this purpose,^107^ for example, by incorporating implementation strategies and outcomes.

In developing the EJDeR logic model, future implementation was not explicitly addressed during the intervention development and feasibility testing phases. While the involvement of public contributors provided valuable insights into contextual factors that may facilitate future implementation, other interest holders (eg, healthcare workers or policymakers) were not included due to limited resources and logistical constraints. Given Sweden is a relatively sparsely populated country with a decentralised healthcare system, it is unclear where EJDeR should be delivered and by whom. Sweden also lacks a dedicated workforce for delivering LICBT interventions.^108^ A structured exploration of implementation barriers and facilitators^109^ in collaboration with key interest holders, such as primary care psychotherapists, may help to better understand the healthcare context and readiness to implement interventions, such as EJDeR.

### Limitations of our programme theory and logic model

Public contributors have been involved throughout intervention development, and their feedback and insights have been integrated into the logic model. However, the actual development of programme theory and logic model was carried out by an ‘expert group’. An alternative approach could have been to include public contributors and healthcare interest holders in a participatory manner, for instance, through theory-of-change workshops.^110^ Further, we did not systematically document the rationale for changes to the logic model during workshops, thereby reducing the transparency of the development process. The need for detailed, structured documentation has been highlighted as essential to improving the transparency of logic model development.^39^

Some concepts, such as intervention acceptability and quality of therapeutic relationship, were challenging to fit into our logic model, due to its structure. We presented the boxes sequentially (except for outputs and mechanisms of change, which are shown to interact reciprocally), but these broad concepts did not fit entirely within a single box. Intervention acceptability (or lack thereof) is present before an individual joins an intervention, may change during the intervention and can also change afterwards.^95^ In other words, acceptability can be both a prerequisite for intervention engagement and be altered by experiences of and outcomes from the intervention. An ongoing process evaluation of EJDeR may provide insight into the relationships among acceptability, the therapeutic relationship and other components of the logic model. As refining and testing programme theory is a core element of the MRC framework,^31^ future evaluations of EJDeR may point to refinements that warrant an updated programme theory and logic model. For example, the current logic model does not include possible adverse events or potential negative mechanisms, which may be captured in future intervention evaluations.

It is important to note that, while logic models help clarify the components of an intervention, they cannot fully capture the dynamic nature of evaluation theories and complex interventions.^111 112^ However, different types of logic models may be appropriate for different purposes and intervention types.^113^ For instance, a more detailed logic model that accounts for context and interconnections may be harder to interpret and may not serve as effectively for communication among interest holders or for reaching consensus on the intervention’s functions. On the other hand, it may be more useful for implementation in new contexts, given the additional information available.

### Future directions

The EJDeR programme theory and logic model continue to be a work in progress, and will be revisited during and after an ongoing randomised controlled pilot trial (RCT) and a future evaluation in a superiority RCT. The pilot RCT aims to address remaining uncertainties regarding the intervention and procedural components (eg, acceptability of randomisation procedures and acceptability of the refined intervention) before progressing to a superiority RCT. In addition, as discussed, future steps towards implementation also need to be taken.

The EJDeR programme theory and logic model was developed by experts and public contributors in isolation from funders, evaluators and healthcare practitioners and future iterations could benefit from engaging a wider range of interest holders. For example, a multisite initiative,^100^ where multiple interest holders collaboratively develop the logic model, could be adopted to include additional perspectives and bridge the gap to implementation.

## Conclusion

We aimed to describe the development of the programme theory and logic model for EJDeR. We have provided an example of how logic model development can be informed by both theory and research, as well as by interest holder feedback, including public contributors. Developing and articulating the programme theory and logic model has clarified the key mechanisms through which the intervention is intended to work. Important contextual factors have been highlighted that may need to be considered for successful implementation or application to new settings. Providing this description may also guide others who wish to develop and articulate the underlying programme theory and logic model of other complex healthcare interventions.

## Data Availability

Data sharing not applicable as no datasets generated and/or analysed for this study.

## References

[1] Svenska Barncancerregistret (SBCR) (2025). Nationellt kvalitetsregister för barncancer. https://sbcr.se/.

[2] Stenmarker M, Mallios P, Hedayati E (2024). Morbidity and mortality among children, adolescents, and young adults with cancer over six decades: a Swedish population-based cohort study (the Rebuc study). *Lancet Reg Health Eur*.

[3] Fardell JE, Wakefield CE, De Abreu Lourenco R (2021). Long-term health-related quality of life in young childhood cancer survivors and their parents. Pediatr Blood Cancer.

[4] Ljungman L, Cernvall M, Grönqvist H (2014). Long-term positive and negative psychological late effects for parents of childhood cancer survivors: a systematic review. PLoS One.

[5] Loecher N, Jordan A, Spunt SL (2024). “You don’t accept he’s completely ok”: a reflexive thematic analysis of parents’ roles in monitoring their child’s health and symptoms after finishing childhood cancer treatment. J Cancer Surviv.

[6] Lundgren J, Thiblin E, Lutvica N (2023). Concerns experienced by parents of children treated for cancer: A qualitative study to inform adaptations to an internet-administered, low-intensity cognitive behavioral therapy intervention. Psychooncology.

[7] Wikman A, Mattsson E, von Essen L (2018). Prevalence and predictors of symptoms of anxiety and depression, and comorbid symptoms of distress in parents of childhood cancer survivors and bereaved parents five years after end of treatment or a child’s death. Acta Oncol.

[8] Carlsson T, Kukkola L, Ljungman L (2019). Psychological distress in parents of children treated for cancer: An explorative study. PLoS One.

[9] Ljungman L, Hovén E, Ljungman G (2015). Does time heal all wounds? A longitudinal study of the development of posttraumatic stress symptoms in parents of survivors of childhood cancer and bereaved parents. Psychooncology.

[10] Kukkola L, Hovén E, Cernvall M (2017). Perceptions of support among Swedish parents of children after end of successful cancer treatment: a prospective, longitudinal study. Acta Oncol.

[11] Hovén E, Grönqvist H, Pöder U (2017). Impact of a child’s cancer disease on parents’ everyday life: a longitudinal study from Sweden. Acta Oncol.

[12] Bakula DM, Sharkey CM, Perez MN (2019). Featured Article: The Relationship Between Parent and Child Distress in Pediatric Cancer: A Meta-Analysis. J Pediatr Psychol.

[13] Moscato E, Patronick J, Wade SL (2022). Family functioning and adaptation following pediatric brain tumor: A systematic review. Pediatr Blood Cancer.

[14] Farrand P, Farrand P (2024). Low-intensity CBT skills and interventions: a practitioner’s manual.

[15] Ljungman L, Boger M, Ander M (2016). Impressions That Last: Particularly Negative and Positive Experiences Reported by Parents Five Years after the End of a Child’s Successful Cancer Treatment or Death. PLoS One.

[16] Ljungman L, Cernvall M, Ghaderi A (2018). An open trial of individualized face-to-face cognitive behavior therapy for psychological distress in parents of children after end of treatment for childhood cancer including a cognitive behavioral conceptualization. PeerJ.

[17] Wikman A, Kukkola L, Börjesson H (2018). Development of an Internet-Administered Cognitive Behavior Therapy Program (ENGAGE) for Parents of Children Previously Treated for Cancer: Participatory Action Research Approach. J Med Internet Res.

[18] Woodford J, Wikman A, Einhorn K (2018). Attitudes and Preferences Toward a Hypothetical Trial of an Internet-Administered Psychological Intervention for Parents of Children Treated for Cancer: Web-Based Survey. JMIR Ment Health.

[19] Woodford J, Farrand P, Hagström J (2021). Internet-Administered Cognitive Behavioral Therapy for Common Mental Health Difficulties in Parents of Children Treated for Cancer: Intervention Development and Description Study. JMIR Form Res.

[20] Reuther C, Lundgren J, Gottvall M (2024). E-therapists’ views on the acceptability and feasibility of an internet-administered, guided, low-intensity cognitive behavioural therapy intervention for parents of children treated for cancer: A qualitative study. Digit Health.

[21] Reuther C, von Essen L, Mustafa MI (2025). Engagement With an Internet-Administered, Guided, Low-Intensity Cognitive Behavioral Therapy Intervention for Parents of Children Treated for Cancer: Analysis of Log-Data From the ENGAGE Feasibility Trial. JMIR Form Res.

[22] Thiblin E, Woodford J, Reuther C (2023). Internet-administered, low-intensity cognitive behavioral therapy for parents of children treated for cancer: A feasibility trial (ENGAGE). Cancer Med.

[23] Thiblin E, Lundgren J, Bergqvist M (2025). The acceptability and feasibility of an internet-administered, guided, low-intensity cognitive behavioural therapy intervention for parents of children treated for cancer: findings from a qualitative study involving public contributors. BMC Psychiatry.

[24] Thiblin E, Reuther C, Bergqvist M (2026). Public Contribution in Qualitative Research With Parents of Children Treated for Cancer: Description of and Reflections on a Collaborative Data Analysis Approach. Health Expect.

[25] Lundgren J, Reuther C, Farrand P (2025). “It is today that counts, and today everything is fine”: coping strategies utilized by parents of children treated for cancer who seek psychological support - a qualitative study. BMC Psychol.

[26] Graneheim UH, Lundman B (2004). Qualitative content analysis in nursing research: concepts, procedures and measures to achieve trustworthiness. Nurse Educ Today.

[27] Gale NK, Heath G, Cameron E (2013). Using the framework method for the analysis of qualitative data in multi-disciplinary health research. BMC Med Res Methodol.

[28] Mellor K, Albury C, Dutton SJ (2023). Recommendations for progression criteria during external randomised pilot trial design, conduct, analysis and reporting. Pilot Feasibility Stud.

[29] Hoddinott P (2015). A new era for intervention development studies. Pilot Feasibility Stud.

[30] Murray J, Baxter R, Lawton R (2023). Unpacking the Cinderella black box of complex intervention development through the Partners at Care Transitions (PACT) programme of research. Health Expect.

[31] Skivington K, Matthews L, Simpson SA (2021). A new framework for developing and evaluating complex interventions: update of Medical Research Council guidance. BMJ.

[32] Bleijenberg N, de Man-van Ginkel JM, Trappenburg JCA (2018). Increasing value and reducing waste by optimizing the development of complex interventions: Enriching the development phase of the Medical Research Council (MRC) Framework. Int J Nurs Stud.

[33] O’Cathain A, Croot L, Duncan E (2019). Guidance on how to develop complex interventions to improve health and healthcare. BMJ Open.

[34] Funnell SC, Rogers PJ (2011). Purposeful program theory: effective use of theories of change and logic models.

[35] Bellg AJ, Borrelli B, Resnick B (2004). Enhancing treatment fidelity in health behavior change studies: best practices and recommendations from the NIH Behavior Change Consortium. Health Psychol.

[36] W.K. Kellogg Foundation (2004). Logic model development guide: using logic models to bring together planning, evaluation, and action.

[37] Rogers PJ, Petrosino A, Huebner TA (2000). Program theory evaluation: practice, promise, and problems. New Drctns Evaluation.

[38] Moore GF, Audrey S, Barker M (2015). Process evaluation of complex interventions: Medical Research Council guidance. BMJ.

[39] Voss S, Bauer J, Coenen M (2025). Logic models for the evaluation of complex interventions in public health: lessons learnt from a staged development process. BMC Public Health.

[40] Williams C, Garland A (2002). A cognitive–behavioural therapy assessment model for use in everyday clinical practice. Adv Psychiatr Treat.

[41] Cuijpers P, Karyotaki E, Harrer M (2023). Individual behavioral activation in the treatment of depression: A meta analysis. Psychother Res.

[42] Cuijpers P, Ciharova M, Tong L (2026). Behavioral activation for depression: A comprehensive systematic review and meta-analysis. Clin Psychol Rev.

[43] Corpas J, Gilbody S, McMillan D (2022). Cognitive, behavioural or cognitive-behavioural self-help interventions for subclinical depression in older adults: A systematic review and meta-analysis. J Affect Disord.

[44] Mazzucchelli TG, Kane RT, Rees CS (2010). Behavioral activation interventions for well-being: A meta-analysis. J Posit Psychol.

[45] Watkins ER, Warren FC, Newbold A (2024). Emotional competence self-help app versus cognitive behavioural self-help app versus self-monitoring app to prevent depression in young adults with elevated risk (ECoWeB PREVENT): an international, multicentre, parallel, open-label, randomised controlled trial. Lancet Digit Health.

[46] Richards DA, Ekers D, McMillan D (2016). Cost and Outcome of Behavioural Activation versus Cognitive Behavioural Therapy for Depression (COBRA): a randomised, controlled, non-inferiority trial. Lancet.

[47] Hopko DR, Lejuez CW, Ruggiero KJ (2003). Contemporary behavioral activation treatments for depression: procedures, principles, and progress. Clin Psychol Rev.

[48] Small F, Lockwood K, Farrand P (2024). Low-Intensity CBT Skills and Interventions: A Practitioner’s Manual.

[49] Peikert ML, Inhestern L, Krauth KA (2022). Fear of progression in parents of childhood cancer survivors: prevalence and associated factors. J Cancer Surviv.

[50] Dugas MJ, Gosselin P, Ladouceur R (2001). Intolerance of uncertainty and worry: investigating specificity in a nonclinical sample. Cognit Ther Res.

[51] Dugas MJ, Ladouceur R (2000). Treatment of GAD. Targeting intolerance of uncertainty in two types of worry. Behav Modif.

[52] Randall CL, McNeil DW (2017). Motivational Interviewing as an Adjunct to Cognitive Behavior Therapy for Anxiety Disorders: A Critical Review of the Literature. Cogn Behav Pract.

[53] Peynenburg V, Hadjistavropoulos H, Thiessen D (2022). Internet-Delivered Cognitive Behavioral Therapy for Postsecondary Students: Randomized Factorial Trial for Examining Motivational Interviewing and Booster Lessons. J Med Internet Res.

[54] Baenziger J, Roser K, Mader L (2020). Post-traumatic stress in parents of long-term childhood cancer survivors compared to parents of the Swiss general population. J Psychosoc Oncol Res Pract.

[55] Pineles SL, Mostoufi SM, Ready CB (2011). Trauma reactivity, avoidant coping, and PTSD symptoms: a moderating relationship?. J Abnorm Psychol.

[56] Wöller W, Mattheß H (2020). TPSS/ROTATE protocol: trauma-focused psycho-social support plus ROTATE. https://www.traumaaid.de/wp-content/uploads/2022/05/TPSS_plus_Trauma-focused_Psycho-Social_Support_plus_ROTATE_English.pdf.

[57] Marsh E, Vallejos EP, Spence A (2022). The digital workplace and its dark side: an integrative review. Comput Human Behav.

[58] Madhav KC, Sherchand SP, Sherchan S (2017). Association between screen time and depression among US adults. Prev Med Rep.

[59] Williams C, Farrand PA, Farrand P (2020). Low-Intensity CBT Skills and Interventions: A Practitioner’s Manual.

[60] Farrand P, Woodford J (2013). Impact of support on the effectiveness of written cognitive behavioural self-help: a systematic review and meta-analysis of randomised controlled trials. Clin Psychol Rev.

[61] Powell CLYM, Chiu CY, Sun X (2024). A meta-analysis on the efficacy of low-intensity cognitive behavioural therapy for generalised anxiety disorder. BMC Psychiatry.

[62] Kuroda N, Burkey MD, Wissow LS (2021). Discovering common elements of empirically supported self-help interventions for depression in primary care: a systematic review. J Gen Intern Med.

[63] Andersson G, Käll A, Juhlin S (2023). Free choice of treatment content, support on demand and supervision in internet-delivered CBT for adults with depression: A randomized factorial design trial. Behav Res Ther.

[64] Mohr DC, Cuijpers P, Lehman K (2011). Supportive accountability: a model for providing human support to enhance adherence to eHealth interventions. J Med Internet Res.

[65] Rushton K, Ardern K, Hopkin E (2020). “I didn’t know what to expect”: Exploring patient perspectives to identify targets for change to improve telephone-delivered psychological interventions. BMC Psychiatry.

[66] Gellatly J, Faija C, Bennion M, Farrand P (2024). Low-Intensity CBT Skills and Interventions: A Practitioner’s Manual.

[67] Waller G, Turner H (2016). Therapist drift redux: Why well-meaning clinicians fail to deliver evidence-based therapy, and how to get back on track. Behav Res Ther.

[68] Drew P, Irvine A, Barkham M (2021). Telephone delivery of psychological interventions: Balancing protocol with patient-centred care. Soc Sci Med.

[69] Small F, Whitworth M, Farrand P (2024). Low-intensity CBT skills and interventions: a practitioner’s manual.

[70] Bennion M, Lovell K, Blakemore A (2025). Predictors of engagement with between-session work in Cognitive Behavioural Therapy (CBT)-based interventions: a mixed-methods systematic review and “best fit” framework synthesis. Cogn Behav Ther.

[71] Bennion M, Blakemore A, Lovell K (2025). Barriers and facilitators to engagement with between-session work for low-intensity Cognitive Behavioural Therapy (CBT)-based interventions: a qualitative exploration of practitioner perceptions. BMC Psychiatry.

[72] Kazantzis N, Whittington C, Zelencich L (2016). Quantity and Quality of Homework Compliance: A Meta-Analysis of Relations With Outcome in Cognitive Behavior Therapy. Behav Ther.

[73] Haller E, Watzke B (2021). The role of homework engagement, homework-related therapist behaviors, and their association with depressive symptoms in telephone-based CBT for depression. Cogn Ther Res.

[74] Cuijpers P, Reijnders M, Huibers MJH (2019). The Role of Common Factors in Psychotherapy Outcomes. Annu Rev Clin Psychol.

[75] Kazantzis N, Dobson KS (2022). Therapeutic relationships in cognitive behavioral therapy: Theory and recent research. Psychother Res.

[76] Thompson M, Parker H, Cave J (2022). Exploring which aspects of a low‐intensity CBT intervention were found to contribute to a successful outcome from the service user point of view: a mixed methods study. Couns and Psychother Res.

[77] Wampold BE, Flückiger C (2023). The alliance in mental health care: conceptualization, evidence and clinical applications. World Psychiatry.

[78] Farrand F, Farrand P (2024). Low-Intensity CBT Skills and Interventions: A Practitioner’s Manual.

[79] Sekhon M, Cartwright M, Francis JJ (2022). Development of a theory-informed questionnaire to assess the acceptability of healthcare interventions. BMC Health Serv Res.

[80] Horvath AO, Greenberg LS (1989). Development and validation of the Working Alliance Inventory. J Couns Psychol.

[81] Hlynsson JI, Sjöberg A, Ström L (2025). Evaluating the reliability and validity of the Questionnaire on Well-Being: a validation study for a clinically informed measurement of subjective well-being. Cogn Behav Ther.

[82] Tennant R, Hiller L, Fishwick R (2007). The Warwick-Edinburgh Mental Well-being Scale (WEMWBS): development and UK validation. Health Qual Life Outcomes.

[83] Kroenke K, Spitzer RL, Williams JB (2001). The PHQ-9: validity of a brief depression severity measure. J Gen Intern Med.

[84] Kroenke K, Strine TW, Spitzer RL (2009). The PHQ-8 as a measure of current depression in the general population. J Affect Disord.

[85] Spitzer RL, Kroenke K, Williams JBW (2006). A brief measure for assessing generalized anxiety disorder: the GAD-7. Arch Intern Med.

[86] Weathers FW, Litz BT, Herman DS (1993). The PTSD Checklist (PCL): reliability, validity, and diagnostic utility.

[87] Gough B, Novikova I (2020). Mental health, men and culture: how do sociocultural constructions of masculinities relate to men’s mental health help-seeking behaviour in the who european region?.

[88] Smith GD, Hebdon M (2024). Mental health help-seeking behaviour in men. J Adv Nurs.

[89] Öhman M, Woodford J, von Essen L (2021). Socioeconomic consequences of parenting a child with cancer for fathers and mothers in Sweden: A population-based difference-in-difference study. Int J Cancer.

[90] Wikman A, Hovén E, Cernvall M (2016). Parents of children diagnosed with cancer: work situation and sick leave, a five-year post end-of-treatment or a child’s death follow-up study. Acta Oncol.

[91] Hollander A-C, Mackay E, Sjöqvist H (2020). Psychiatric care use among migrants to Sweden compared with Swedish-born residents: a longitudinal cohort study of 5 150 753 people. BMJ Glob Health.

[92] Nwokoroku SC, Neil B, Dlamini C (2022). A systematic review of the role of culture in the mental health service utilisation among ethnic minority groups in the United Kingdom. Glob Ment Health (Camb).

[93] Salomonsson S, Santoft F, Lindsäter E (2020). Predictors of outcome in guided self-help cognitive behavioural therapy for common mental disorders in primary care. Cogn Behav Ther.

[94] Cuijpers P, Harrer M, Miguel C (2026). Does baseline severity interact with the effects of psychotherapy for depression? A meta-analytic review. J Affect Disord.

[95] Sekhon M, Cartwright M, Francis JJ (2017). Acceptability of healthcare interventions: an overview of reviews and development of a theoretical framework. BMC Health Serv Res.

[96] Inhestern L, Nasse ML, Krauth KA (2024). Reintegration into school, kindergarten and work in families of childhood cancer survivors after a family-oriented rehabilitation program. Front Pediatr.

[97] Kälin S, Baenziger J, Mader L (2025). Health-related quality of life in parents of long-term childhood cancer survivors: a report from the Swiss Childhood Cancer Survivor Study - Parents. Qual Life Res.

[98] Barncancerfonden (2024). Mitt barn är färdigbehandlat – sen då? Information till föräldrar. https://www.barncancerfonden.se/globalassets/bilder/for-drabbade/informationsmaterial/mitt-barn-ar-fardigbehandlat_juni2024.pdf.

[99] Ludvigsson JF, Bergman D, Lundgren CI (2025). The healthcare system in Sweden. Eur J Epidemiol.

[100] Easterling DV, Jacob RR, Brownson RC (2023). Participatory logic modeling in a multi-site initiative to advance implementation science. *Implement Sci Commun*.

[101] Naeem F (2019). Cultural adaptations of CBT: a summary and discussion of the Special Issue on Cultural Adaptation of CBT. tCBT.

[102] Spatuzzi R, Giulietti MV, Ricciuti M (2019). Exploring the associations between spiritual well-being, burden, and quality of life in family caregivers of cancer patients. Palliat Support Care.

[103] Chong ASS, Ahmad MB, Alias H (2023). Spiritual Coping Among Families of Children With Cancer: A Qualitative Study. Asia Pac J Public Health.

[104] Treweek S, Gillies K, Witham MD (2024). How should trial teams make decisions about the proportions and diversity of the ethnic groups in their trial?. Trials.

[105] Moore G, Campbell M, Copeland L (2021). Adapting interventions to new contexts-the ADAPT guidance. BMJ.

[106] Moullin JC, Dickson KS, Stadnick NA (2020). Ten recommendations for using implementation frameworks in research and practice. *Implement Sci Commun*.

[107] Smith JD, Li DH, Rafferty MR (2020). The Implementation Research Logic Model: a method for planning, executing, reporting, and synthesizing implementation projects. Implement Sci.

[108] Brantnell A, Woodford J, Baraldi E (2020). Views of Implementers and Nonimplementers of Internet-Administered Cognitive Behavioral Therapy for Depression and Anxiety: Survey of Primary Care Decision Makers in Sweden. J Med Internet Res.

[109] Damschroder LJ, Reardon CM, Widerquist MAO (2022). The updated Consolidated Framework for Implementation Research based on user feedback. Implement Sci.

[110] Abayneh S, Lempp H, Alem A (2020). Developing a Theory of Change model of service user and caregiver involvement in mental health system strengthening in primary health care in rural Ethiopia. Int J Ment Health Syst.

[111] Miller RL (2013). Logic models: a useful way to study theories of evaluation practice?. Eval Program Plann.

[112] Greenwood-Lee J, Hawe P, Nettel-Aguirre A (2016). Complex intervention modelling should capture the dynamics of adaptation. BMC Med Res Methodol.

[113] Mills T, Lawton R, Sheard L (2019). Advancing complexity science in healthcare research: the logic of logic models. BMC Med Res Methodol.

