# Supplementary material for: Development and description of a logic model for a guided, low-intensity cognitive behavioural therapy intervention for parents of persons treated for cancer during childhood: the EJDeR intervention
Source: BMJ Open. 2026 Oct 13;16(10):e116503. doi: 10.1136/bmjopen-2026-116503 (PMC13663751; doi:10.1136/bmjopen-2026-116503)
Supplement: Supplementary file 1 [file bmjopen-2026-116503.reviewer_comments.pdf]

PEER REVIEW HISTORY

BMJ Open publishes all reviews undertaken for accepted manuscripts. Reviewers are asked to complete a checklist review form and are provided with free text boxes to elaborate on their assessment. These free text comments are reproduced below.

ARTICLE DETAILS

Title (Provisional)

Development and description of a logic model for a guided, low-intensity cognitive behavioural therapy intervention for parents of persons treated for cancer during childhood: The EJDeR intervention

Authors

Thiblin, Ella; Woodford, Joanne; Berman, Anne H.; Farrand, Paul; Leissner, Philip; Saarijärvi, Markus; Von Essen, Louise; Richards, David A

VERSION 1 - REVIEW

**Reviewer**	**1**
**Name**	**Bhavsar, Vishal**
**Affiliation**	**King's College London, Department of Health Services and Population Research**
**Date**	**25-Jun-2026**
**COI**	**None**

Thank you for this very readable and clear description of development of a complex intervention theory for an important clinical problem. My main suggestion is that given the journal and audience, the amount of spice given over to justifying the use of the MRC framework at the outset seems out of place and removing this to focus on the clinical problem from the outset would make for a much more focused work. I think justification of the approach could be done much more efficiently (e.g. in the methods) than currently. This somewhat detracts in my view from the clinical problem itself.

For example, could the abstract background include information on the clinical problem of childhood cancer? I appreciate the methodological emphasis, but saying that you applied the framework to families affected by cancer in this section would be helpful.

There is a typo at LIMIATIONS on page 3.

There is clearly a contribution to intervention knowledge here in the filed of cancer affecting children. I am less convinced personally about the emphasis on aligning with MRC guidance- this is very widely cited and endorsed guidance and the consideration of its integration into

your research here is not clearly articulated as a novel contribution in itself, and I wonder if a more streamlined version of this would simply identify the problem, highlight the complexity and suitability of MRC/NIHR as an organizing framework, and focus on the intervention itself. In line with my personal preference for a shorter paper which focuses on the intervention itself rather advancing/justifying the methodological framework, this might also be aided by closer editing: there is repetition in the Conclusion of “we will continue to refine the model” which is stated in the “Future directions” section.

Could you review:” The EJDeR programme theory and logic model was informed by public contributors but was developed by experts in isolation from funders, evaluators, and health-care practitioners. For example, a multi-site initiative [see 96] could be adopted to address these areas to further bridge the gap to implementation” – this passage was unclear to me, especially as to how the two sentences link together?

The diagrams are clear and appropriate – the authors might consider identifying programme assumptions in the logic model diagram, as well as in the text.

**Reviewer**	**2**
**Name**	**Hoge, Margaret**
**Affiliation**	**UT Southwestern**
**Date**	**29-Jul-2026**
**COI**	**None**

This is a descriptive paper on the iterative process of creating a behavioral intervention to help parents of children with a cancer diagnosis. It seems to follow the ORBIT model for creating a behavioral intervention using mixed methods both survey responses and qualitative interviews to obtain knowledge to create the intervention and test it in the future for an RCT.

Strengths:

- This is an important product to publish to allow other researchers to know how to follow a similar process to create complex behavioral interventions.

Weaknesses/Improvements:

- I believe it would be helpful for the reader in the introduction to include the potential mechanism of action before the creation of the logic. For example, discuss that parents of children diagnosed with cancer have X amount of depression, anxiety, and PTSD which impacts the parents’ thoughts, feelings, and behaviors towards their children. By targeting the mechanism of parental mental health symptoms, the hypothesis is that a behavioral intervention can be created using stakeholder input to improve parent mental health and interactions with their children.

-It was unclear the plan for the qualitative analysis or preparation, such as interview creation and software used for coding and thematic analysis. It was also unclear the thematic analysis results to help inform the next phases of the intervention creation.

-It was unclear what surveys were used and the results of these surveys to help inform the next phase.

- It was unclear the timing of the intervention. Is it during the active cancer treatment or afterwards when the child is in remission? The stressors and responses would be different in these time periods and important to distinguish.

- The figures were wordy and had to follow. They would benefit from being condensed and less verbose.

- It was unclear the inclusion and exclusion criteria for what parents and stakeholders would be approached and consented.

- It was unclear the IRB approval for this study/intervention creation.

Strengths:

- This is an important product to publish to allow other researchers to know how to follow a similar process to create complex behavioral interventions.

Weaknesses/Improvements:

- I believe it would be helpful for the reader in the introduction to

---

VERSION 1 - AUTHOR RESPONSE

Reviewer: 1

Dr. Vishal Bhavsar, King's College London, King's College London

Comments to the Author:

Thank you for this very readable and clear description of development of a complex intervention theory for an important clinical problem.

Thank you for reviewing our manuscript and for your helpful comments.

1. My main suggestion is that given the journal and audience, the amount of spice given over to justifying the use of the MRC framework at the outset seems out of place and removing this to focus on the clinical problem from the outset would make for a much more focused work. I think justification of the approach could be done much more efficiently (e.g. in the methods) than currently. This somewhat detracts in my view from the clinical problem itself.

For example, could the abstract background include information on the clinical problem of childhood cancer? I appreciate the methodological emphasis, but saying that you applied the framework to families affected by cancer in this section would be helpful.

Thank you for this suggestion. We understand that your main feedback concerns the paper's emphasis, and you would prefer a focus on the clinical problem rather than the methodology. We appreciate this input and have restructured our paper to start with the clinical problem. However, we find it helpful to

include an introduction to the core element of programme theory in the MRC Framework, as we have had difficulty finding clear guidance on this in the literature. We have therefore kept some background to the MRC Framework in the introduction, but in a shortened form that we hope will be more concise.

As per your suggestion, we have included information on the population earlier in the abstract, see page 2:

> **ABSTRACT**
>
> **Background**. There is a lack of psychological interventions for parents of persons treated for cancer during childhood who report a need for support. We therefore developed a psychological intervention, including a programme theory and logic model as recommended by the United Kingdom Medical Research Council. The logic model offers structured ways to articulate how interventions are intended to work and interact within their context. The processes by which programme theories and logic models are developed and refined are rarely described in detail.

2. There is a typo at LIMIATIONS on page 3.

Thank you for noticing. We have corrected the typo.

3. There is clearly a contribution to intervention knowledge here in the filed of cancer affecting children. I am less convinced personally about the emphasis on aligning with MRC guidance- this is very widely cited and endorsed guidance and the consideration of its integration into your research here is not clearly articulated as a novel contribution in itself, and I wonder if a more streamlined version of this would simply identify the problem, highlight the complexity and suitability of MRC/NIHR as an organizing framework, and focus on the intervention itself. In line with my personal preference for a shorter paper which focuses on the intervention itself rather advancing/justifying the methodological framework, this might also be aided by closer editing: there is repetition in the Conclusion of "we will continue to refine the model" which is stated in the "Future directions" section.

Thank you for this comment. As stated in our response to your first comment, we appreciate the opportunity to streamline the paper and put the clinical problem and intervention in the spotlight, rather than the MRC Framework. We hope that the changes mentioned, with a different order of presentation and a condensed description of programme theories and logic models, align the paper with your suggestions.

We have now omitted the repetition of "We will continue to refine the programme theory and logic model in future intervention testing and evaluation" from the conclusions section.

4. Could you review:" The EJDeR programme theory and logic model was informed by public contributors but was developed by experts in isolation from funders, evaluators, and health-care practitioners. For example, a multi-site initiative [see 96] could be adopted to address these areas to further bridge the gap to implementation" – this passage was unclear to me, especially as to how the two sentences link together?

Thank you for pointing out that this passage may be difficult to follow. We aim to convey that there are structured methods for engaging additional interest holders in programme theory and logic model development that the EJDeR logic model could benefit from adopting. We have rephrased, and hope that our point is now made clearer, see page 22:

> The EJDeR programme theory and logic model was developed by experts and public contributors in isolation from funders, evaluators, and health-care practitioners, and future iterations could benefit from engaging a wider range of interest holders. For example, a multi-site initiative [100], where multiple interest holders collaboratively develop the logic model, could be adopted to include additional perspectives and bridge the gap to implementation.

5. The diagrams are clear and appropriate – the authors might consider identifying programme assumptions in the logic model diagram, as well as in the text.

Thank you for this suggestion. However, Reviewer 2 suggested condensing the figures, and we do see that the logic model may be perceived as too detailed in its current form. After consideration, we have decided not to add programme assumptions, but we will continue to reflect on what to include for future iterations of the logic model.

Reviewer: 2
Dr. Margaret Hoge, UT Southwestern
Comments to the Author:
This is a descriptive paper on the iterative process of creating a behavioral intervention to help parents of children with a cancer diagnosis. It seems to follow the ORBIT model for creating a behavioral intervention using mixed methods both survey responses and qualitative interviews to obtain knowledge to create the intervention and test it in the future for an RCT.

Strengths:
- This is an important product to publish to allow other researchers to know how to follow a similar process to create complex behavioral interventions.

Thank you for reviewing our paper and for your constructive comments and suggestions. For readability, we have numbered your comments and provided responses to them separately.

Weaknesses/Improvements:
1. I believe it would be helpful for the reader in the introduction to include the potential mechanism of action before the creation of the logic. For example, discuss that parents of children diagnosed with cancer have X amount of depression, anxiety, and PTSD which impacts the parents' thoughts, feelings, and behaviors towards their children. By targeting the mechanism of parental mental health symptoms, the hypothesis is that a behavioral intervention can be created using stakeholder input to improve parent mental health and interactions with their children.

Thank you for this suggestion. We have restructured the introduction overall in response to both your and Reviewer 1's comments. Now, the clinical problem is introduced first, and we have added detail on the symptoms and mechanisms of the problem, as per your comment, see page 3-4:

> Challenges such as fatigue, depressive symptoms, worry, uncertainty about the future, and feelings of isolation are reported by parents, and can persist after completion of the child's cancer treatment [3-8]. Across studies, symptoms of depression and anxiety reported by parents ranged from 14.4% to 43.4% and from 19.7% to 43.4%, respectively [3, 7]. Five years after completion of cancer treatment, post-traumatic stress symptoms were reported by 7.8% of fathers and 19.1% of mothers [9]. In Sweden, a need for psychological support is reported by 37% of mothers and 28% of fathers one year after treatment completion, and 19% of mothers and 7% of fathers five years after treatment [10]. Difficulties with psychological well-being may affect

> parents' everyday life (e.g., leisure activities and work) [11]. Furthermore, increased parental psychological distress is associated with greater distress in the child treated for cancer [12], and overall family functioning supports healthy psychosocial development in the child [13]. Therefore, it is important to provide evidence-based care and treatment for parents to improve their mental well-being and, by extension, overall family functioning and the child's well-being.

2. It was unclear the plan for the qualitative analysis or preparation, such as interview creation and software used for coding and thematic analysis. It was also unclear the thematic analysis results to help inform the next phases of the intervention creation.

Thank you for this comment. We have outlined the methods and results for the qualitative interviews in detail in separate papers. We have now clarified this and provided some basic information on the qualitative interviews and how results were used, see page 5:

> We collected clinical and procedural data and conducted qualitative interviews at baseline to examine parents' cancer-related concerns and coping strategies [6, 25]. We conducted interviews at post-treatment (12 weeks) to explore the acceptability of EJDeR from the perspective of parents [23] and parent guides [20]. Interviews were analysed with qualitative content analysis [26] and the framework method [27], (using NVivo software). Results subsequently informed modifications and refinements to EJDeR, e.g., including additional psychoeducation and refining intervention language.

3. It was unclear what surveys were used and the results of these surveys to help inform the next phase.

RESPONSE: Thank you for this comment. Survey results have been reported separately, and we have now included a clearer reference to that publication, see page 4:

> A summary of studies conducted during these phases, leading up to intervention refinements and the programme theory and logic model development, is outlined in Figure 1, including preparatory longitudinal work [9-11, , a systematic review [4], a survey on attitudes and preferences towards an intervention [18], and feasibility testing [6, 20-25].

4. It was unclear the timing of the intervention. Is it during the active cancer treatment or afterwards when the child is in remission? The stressors and responses would be different in these time periods and important to distinguish.

Thank you for pointing out this unclarity. We have now clarified that the intervention targets parents of children who have ended cancer treatment, see page 4:

> The goal of EJDeR is to improve mental well-being and reduce psychological distress among parents of persons who have completed treatment for cancer during childhood three months to five years previously, and are in remission
> -

5. The figures were wordy and had to follow. They would benefit from being condensed and less verbose.

Thank you for this comment. We agree that some of the figures benefit from condensation. We have condensed the text in Figures 2 and 4 slightly to aid comprehension, whilst retaining the necessary structure and details. For instance, we have reduced the number of outcomes included in Figure 2, and

collapsed bullet points in Figure 4 (e.g., "Modules offered" under Intervention Delivery rather than listing "X module offered" as separate points). Regarding Figure 3, this is an intermediate version of the logic model we subsequently reworked into the one in Figure 4, as we found the intermediate model too detailed for effective communication with external interest holders. We have therefore decided to keep it as it is to illustrate that one may have to make different iterations of a logic model before arriving at a version that balances information with readability. We have clarified this rationale for including the figure in the text, see page 8:

> An intermediate version of the logic model is shown in Figure 3. During this stage, we decided to use a logic model typology outlining intervention resources/input, intervention delivery/activities, intervention outputs, mechanisms of impact, outcomes, and context. This iteration was found to be too complex and detailed for effective communication with interest holders outside the research team.

And page 9:

> The final logic model is shown in Figure 4. It has a simpler structure, and includes input from public contributors, e.g., the mode of delivery was adapted to provide a workbook and an audio-administered version of EJDeR, and contextual factors, e.g., the child's diagnosis and treatment, and parents' prior experiences with healthcare were added.

6. It was unclear the inclusion and exclusion criteria for what parents and stakeholders would be approached and consented.

Thank you for this comment! We have now provided a brief description of public contributors, with clear references to publications where inclusion and exclusion criteria are outlined, see page 4:

> Public contributors in the intervention development phase were four parents (2 fathers and 2 mothers) of persons treated for cancer during childhood [19] , and in the feasibility phase three former ENGAGE participants (2 fathers and 1 mother) [23, 24], and three parents (2 mothers and 1 father) of persons treated for cancer during childhood who did not participate in ENGAGE.

7. It was unclear the IRB approval for this study/intervention creation.

Thank you for noticing! An ethical approval statement has now been added to the main document, in addition to the submission system where it was originally included, see page 2:

> **Ethical approval statement**
>
> Ethical approval for studies informing the EJDeR intervention development process was granted by the regional ethical review board in Uppsala (DNR: 2012/440; DNR: 2015/426; DNR: 2017/527) and the Swedish Ethical Review Authority (DNR: 2019-03083). The ENGAGE feasibility trial was approved by the Regional Ethical Review Board in Uppsala, Sweden (Dnr: 2017/527), and conducted in accordance with the Declaration of Helsinki, ensuring the welfare and rights of all participants, as well as Good Clinical Practice (GCP) guidelines. Ethical approval for forming the Parent Advisory Board was obtained from the Swedish Ethical Review Authority (Dnr:2023-06850-01).

VERSION 2 - REVIEW

Reviewer 1
Name Bhavsar, Vishal
Affiliation King's College London, Department of Health Services and Population Research
Date 23-Sep-2026
COI

Thank you I think this is much improved. I would encourage the authors to highlight the patient population/clinical problem higher up in the paper/Introduction. I appreciate the authors' engagement with my comments so far.

VERSION 2 - AUTHOR RESPONSE

Reviewer: 1
Dr. Vishal Bhavsar, King's College London, King's College London
Comments to the Author:
Thank you I think this is much improved. I would encourage the authors to highlight the patient population/clinical problem higher up in the paper/Introduction. I appreciate the authors' engagement with my comments so far.

Thank you for your feedback and additional comment. We have now introduced the population and clinical problem in the first sentence of the introduction, see page 3:

> Parents of persons treated for cancer during childhood often experience significant psychological challenges. Approximately 350 children are diagnosed with cancer in Sweden each year [1]. As cancer treatments have advanced, the five-year survival rate for children and adolescents with cancer in Sweden is now 85% [2]. During and after treatment, parents are typically the main support for children with cancer, and a diagnosis of childhood cancer is a highly disruptive experience for children and parents. Challenges such as fatigue, depressive symptoms, worry, uncertainty about the future, and feelings of isolation are reported by parents, and can persist after completion of the child's cancer treatment [3-8]. Across studies, symptoms of depression and anxiety reported by parents ranged from 14.4% to 43.4% and from 19.7% to 43.4%, respectively [3, 7]. Five years after completion of cancer treatment, post-traumatic stress symptoms were reported by 7.8% of fathers and 19.1% of mothers [9]. In Sweden, a need for psychological support is reported by 37% of mothers and 28% of fathers one year after treatment completion, and 19% of mothers and 7% of fathers five years after treatment [10]. Difficulties with psychological well-being may affect parents' everyday life (e.g., leisure activities and work) [11]. Furthermore, increased parental psychological distress is associated with greater distress in the child treated for cancer [12], and overall family functioning supports healthy psychosocial development in the child [13]. Therefore, it is important to provide evidence-based care and treatment for parents to improve their mental well-being and, by extension, overall family functioning and the child's well-being.
